# LY86 facilitates ox-LDL-induced lipid accumulation in macrophages by upregulating SREBP2/HMGCR expression

**DOI:** 10.1186/s12872-024-03957-1

**Published:** 2024-05-31

**Authors:** Guangwei Jiang, Jikuan Li, Shuai Niu, Ruoyu Dong, Yuyan Chen, Wei Bi

**Affiliations:** 1 Department of Vascular Surgery, The Second Hospital of Hebei Medical University, Shijiazhuang, 050000 China; 2 Department of Vascular Surgery, Hebei General Hospital, Shijiazhuang, 050000 China; 3 The Second Department of rehabilitation Medicine, The Second Hospital of Hebei Medical University, Shijiazhuang, 050000 China

**Keywords:** LY86, ox-LDL, Lipid accumulation, SREBP2/HMGCR, Macrophages

## Abstract

LY86, also known as MD1, has been implicated in various pathophysiological processes including inflammation, obesity, insulin resistance, and immunoregulation. However, the role of LY86 in cholesterol metabolism remains incompletely understood. Several studies have reported significant up-regulation of LY86 mRNA in atherosclerosis; nevertheless, the regulatory mechanism by which LY86 is involved in this disease remains unclear. In this study, we aimed to investigate whether LY86 affects ox-LDL-induced lipid accumulation in macrophages. Firstly, we confirmed that LY86 is indeed involved in the process of atherosclerosis and found high expression levels of LY86 in human atherosclerotic plaque tissue. Furthermore, our findings suggest that LY86 may mediate intracellular lipid accumulation induced by ox-LDL through the SREBP2/HMGCR pathway. This mechanism could be associated with increased cholesterol synthesis resulting from enhanced endoplasmic reticulum stress response.

**Supplementary Information:**

The online version contains supplementary material available at 10.1186/s12872-024-03957-1.

## Introduction

The global prevalence of cardiovascular disease leading to mortality is progressively increasing [1]. As widely acknowledged, atherosclerosis arises from multiple risk factors encompassing hyperlipidemia, hypertension, diabetes, and smoking [2]. Chronic inflammation represents a hallmark of atherosclerosis; vascular inflammation can be mediated by bioactive lipids, proinflammatory cytokines, and adhesion molecules in a persistent manner [3]. In the presence of cholesterol-rich lipoproteins within the arterial wall, circulating monocytes are recruited and differentiate into macrophages [4]. Macrophages, through their surface scavenger receptors, possess the ability to phagocytize oxidized LDL (ox-LDL) in substantial quantities, secrete pro-inflammatory cytokines and ROS [5], thereby exacerbating oxidative lipid modification [6]. Given that macrophage transformation into foam cells is a crucial element of atherosclerotic lesion growth [7], these cells play a pivotal role in this process.

Lymphocyte antigen 86 (LY86), also known as myeloid differentiation protein 1 (MD1), encodes a secreted glycoprotein that participates in inflammation, humoral immune response, apoptosis, and signal transduction [8]. LY86 is widely expressed in cardiac tissues and predominantly by B cells and macrophages [9–11]. Recent studies have demonstrated that the negative role of LY86 in various cardiac pathologies such as pressure overload-induced cardiac and electrical remodeling and high-fat diet-induced atherosclerosis [10, 12]; furthermore, LY86 deficiency exacerbates cardiac hypertrophy [10].

Xu et al. identified LY86 as an up-regulated gene in atherosclerosis through analysis of the DEGs database. LY86 encodes a crucial cell membrane protein involved in transmembrane signal transduction [13]. Subsequently, using the PPI network, LY86 was identified as a key DEG that exhibited up-regulation in the carotid arteries of mice for 7 days after ligation [14]. However, the precise role of LY86 in atherosclerosis remains incompletely understood. In our study, we collected common carotid artery plaque tissues and observed an up-regulation of LY86 expression in atherosclerosis. This finding was further confirmed in ApoE -/- mice fed with a high-fat diet (HFD) for 8 weeks, which aligned with the results obtained from human carotid artery plaque samples. By treating THP-1 monocytic cells with phorbol myristate acetate (PMA), we successfully differentiated them into macrophages [15]. Subsequently, upon knocking down LY86 in these macrophages and stimulating them with ox-LDL, we observed reduced lipid accumulation and lower oxidative stress levels [16].

## Materials and methods

### Clinical samples and data collection

Patients admitted to the Department of Vascular Surgery at Hebei General Hospital between January 2023 and June 2023 for severe carotid artery stenosis or lower limb varicose veins were retrospectively analyzed. Patients aged between 18 and 80 years old, with carotid artery severe stenosis or lower limb venous curvature who underwent surgical treatment, volunteered for this survey, and had complete case data were included in the study. Patients with liver or kidney dysfunction, hereditary hyperlipidemia, the use of drugs that may affect lipid metabolism levels, malignant neoplasms disease and hyperthyroidism were excluded from the study. The patients were categorized into two groups based on their disease: the arteriosclerosis group(AS group) and the non-arteriosclerosis group(Non-AS group). All patient were instructed to adhere to an 8-hour fasting period prior to specimen collection upon initial hospitalization. A volume of 5 ml blood was obtained from the cubital vein, and serum lipid levels were quantified using an automated biochemical analyzer.The demographic data and serum lipid levels of the patients were subjected to statistical analysis. The carotid plaque and adjacent normal intimal tissue were obtained from patients who underwent carotid endarterectomy. The AS group consisted of carotid plaque samples, while adjacent normal intimal tissue was utilized as the control group.

### Experimental animals

The male 10 ApoE^−/−^ and 10 C57BL/6J mice (8weeks, SPF grade) were purchased from Changsheng Biotechnology Co., Ltd, (Liaoning, China, license number: SCXK (Liao) 2020-0001). All mice were raised at the Hebei General Hospital Animal Center. The breeding conditions are a light-controlled (12 h: 12 h light/dark alter) room with temperature 22 ± 1 °C and humidity 40-45%.

### Model preparation and sample collection

After one week of adaptive rearing with regular rodent chow. The C57BL/6J mice were used as the control group (ND group), and the ApoE^−/−^ mice were used as the model group (HFD group). The ApoE^−/−^ mice were fed high-fat diet (ingredients: protein 24.2%, carbohydrates 40.1%, fat 27.4%, cholesterol 2%, total calorie ratio 4.7 kcal/gm) to establish the AS model, while the ND group was fed with regular rodent chow. The two groups mice were fed with the same quality of feed every day. After 8 weeks, the mice were anesthetized with 3% pentobarbital sodium after fasting overnight. Then, after weighing and collecting blood from the inner canthus, physiological saline was used to perfuse the left ventricle of mice, and the mice were sacrificed by cervical dislocation under anesthesia. Afterwards, the entire aorta were quickly dissected for subsequent detection of relevant indicators.

### Cell processing and grouping

The THP-1 cell line was obtained from Starfish Biologicals and cultured in 1640 complete medium supplemented with 10% FBS and 1% PS at 37 °C in a CO2 incubator (5%). For well plate inoculation experiments, THP-1 cells in the logarithmic growth phase were pre-induced into macrophages using PMA (500 ng/mL). The experimental groups included the blank control group, ox-LDL stimulation group, si-LY86 knockdown group, and si-LY86 knockdown + ox-LDL stimulation group. In all experiments, LY86 sequence was knocked down for 24 h followed by stimulation with ox-LDL (50 µg/mL) for another 24 h (siRNA from Reebok Biotech; ox-LDL from Yiyuan Biotech, YB-002).

### Lipid parameters

The blood samples were centrifuged at 4 °C and 3000r/min for 15 min to obtain the supernatant. The levels of total cholesterol (T-CHO, batch A111-1-1 ), triglycerides (TG, batch A110-1-1), high density lipoprotein cholesterol (HDL-C, batch A112-1-1), and low density lipoprotein cholesterol (LDL-C, batch A113-1-1) were determined by a automatic biochemical analyzer (7600–020, Hitachi, Tokyo, Japan).

### Immunohistochemistry and Immunofluorescence

The human tissues were embedded in paraffin and sectioned at a thickness of 5 μm. Following dewaxing and hydration, some tissues underwent HE staining, while the remaining tissue sections were subjected to LY86 staining after antigen retrieval with sodium citrate. False positives were minimized by dropwise addition of an appropriate amount of endogenous peroxidase blocker. The primary antibody (LY86, 1:10, Santa Cruz Biotechnology, F-5) was added dropwise and incubated at 37℃ for 1 h. Subsequently, reaction enhancement solution was added dropwise and incubated at 37℃ for 20 min. Enhanced enzyme-labeled goat anti-mouse/rabbit IgG polymers were then added dropwise and incubated at 37℃ for 20 min followed by DAB color development and restaining with hematoxylin. Finally, the slices were sealed, photographed using a microscope, and statistically analyzed using the Histochemistry Kit from ZSGB-BIO.

For immunofluorescence analysis, the aortic arch of mice were embedded in OCT and sectioned at a thickness of 4 μm. The tissue sections were washed with PBS for three times. Then the slices were permeabilized with 0.2% Triton X-100 and blocked in 5% normal donkey serum for 1 h and stained with primary antibody overnight. Next, the slices were washed with PBS for three times and incubated with the fluorescent-conjected secondary antibody for 1 h at room temperature. PBS was used to wash the slices for three times before using DAPI to stain the nucleus. Primary antibodies were: anti- LY86 (dilution: 1:50, Santa Cruz Biotechnology, C2918), anti- HMGCR (dilution: 1:100, PTMab PTM-6018), anti-CD68 (dilution: 1:100, Proteintech, 28058-1-AP), anti-GRP78 (dilution: 1:100, Proteintech, 11587-1-AP). Finally, the slices were observed with an Olympus IX71 fluorescence microscope (Olympus, Japan).

### Western blots

Total proteins were extracted from human tissues or THP-1 cells treated with NP-40 lysate (Bohazel Bio). The protein samples were resolved using 10% SDS-PAGE fast gel (meilunbio) and transferred onto methanol-activated PVDF membranes (Merck & Millipore). Subsequently, the membranes were blocked with 5% skimmed milk for 1 h, followed by overnight incubation at 4℃ with the primary antibodies. Afterward, HRP-coupled goat anti-mouse or rabbit IgG (Santa Cruz Biotechnology) was applied to the membrane for 1 h at room temperature. Protein bands were visualized using an ECL chemiluminescence kit and captured using an imaging system. The gray values of the protein bands were quantified utilizing Image Lab software. The primary antibodies used in this study included: anti-LY86 (dilution: 1:500, Santa, C2918), anti-SREBP2 (dilution: 1:1000, Affinity, AF0450), anti-β-actin (dilution: 1:10000, Abclonal, AC026), anti-GRP78 (dilution: 1:1000, Affinity, AF5366), and anti-HMGCR (dilution: 1:1000, PTMab PTM-6018), anti-ABCA1 (dilution: 1:1000, HUABIO, R1510-42), anti-CD86 (dilution: 1:1000, Proteintech, 26903-1-AP), anti-CD68 (dilution: 1:500, Affinity, DF7518), anti-MMP9 (dilution: 1:600, Affinity, AF5228).

### Cell transfection experiments

The THP-1 cells were seeded in six-well plates, and once the cells adhered to the surface, the medium was replaced with 1640 complete medium supplemented with 2% FBS. Complexes of siRNA (5 µL siRNA + 200 µL 1640 basal medium + 5 µL HighGene) were prepared in sterile, enzyme-free EP tubes and added to the well plates to achieve a final siRNA concentration of 50 nM. Half of the medium was exchanged after 5 h, and the knockdown effect was assessed by Western blotting and qRT-PCR analysis after 24 h.

### Quantitative real-time polymerase chain reaction (qRT-PCR) assay

The RNA was extracted from human tissues or group-treated cells using Trizol reagent (TAKAZA). The concentration was determined using the microplate method, followed by reverse transcription of the appropriate amount of RNA (Vazyme, HiScript ® III RT SuperMix for qPCR, R323). Subsequently, the cDNA was amplified in the X960 system (Sangon Biotech, SGExcel FastSYBR qPCR, B532955). The primer sequence used for LY86 amplification were as follows: Forward - GTTTCACAGCCACTCTCTTCCTC and Reverse – TTGTAATGGATCGCAACTCT -GGTAG. For quantitative analysis, β-actin served as an internal reference and 2^−△△CT^ values were calculated for statistical analysis.

### Oil red o staining

The oil red O storage solution was prepared by dissolving 1 g of oil red O powder in 100 mL of isopropanol. Subsequently, the oil red O storage solution was diluted and mixed with double-distilled water at a ratio of 3:2. After filtering impurities using qualitative filter paper under dark conditions, the solution was prepared for use. The cells and entire aorta were fixed with 4% paraformaldehyde for 10 min and washed twice with PBS. For cells, each well was treated with 1 mL of oil red O staining solution for 30 min. After discarding the staining solution, the cells were washed repeatedly with double-distilled water until the liquid became colorless and stained with hematoxylin. Finally, lipid droplet staining was observed under a microscope to confirm successful staining. The entire aorta was stained with oil red O for 30 min, and then removed non-specific staining with 60% isopropanol. Cut the blood vessels along the midline and captured a tile image.

### Data analysis

Statistical analysis was conducted using SPSS software (version 26.0, IBM, Chicago, IL, USA). The distribution of quantitative data was assessed using the Shapiro-Wilk test. Mean ± SD was used to present normally distributed data and compared between groups through an independent sample t-test or One-way ANOVA. Non-normally distributed measurement data were represented as *M* (*Q1*, *Q3*), and group comparisons were made using the Wilcoxon test. Qualitative data were expressed as numbers and percentages (n%) and analyzed for group differences with Fisher’s exact probability test or Pearson’s chi-square test. The criterion for statistical significance was set at *P* < 0.05.

## Result

### Basic clinical data of normal group and atherosclerosis patient group

We conducted a retrospective analysis of 689 patients, among whom 87 patients met the criteria. Specifically, 67 patients were classified into the AS group while the remaining 20 patients belonged to the Non-AS group. There were no statistically significant differences observed in terms of gender, age, BMI, comorbidities, and risk factors between the two groups (*P* > 0.05) (Table 1). In comparison to the Non-AS group, the AS group exhibited significantly higher levels of serum T-CHO, TG, LDL-C, very-low-density lipoprotein (VLDL), and lipoprotein(a) [LP(a)]. However, there were no significant differences observed in terms of HDL-C, apolipoprotein A1(ApoA1) and apolipoprotien B(ApoB) levels between the two groups(Table 2).

**Table 1:** General data between these two groups of patients

Variables	AS(*n* = 67)	Non-AS(*n* = 20)^a^	*P*-value
Age, year	65.2 ± 8.5	61.4 ± 5.7	0.067
Men	54	6	0.314
BMI, kg/m²	25.9 ± 2.9	25.3 ± 3.4	0.427
Co-morbidities, n			
Hypertension	46	10	0.126
Diabetes mellitus	17	4	0.622
Coronary heart disease Ischemic stroke	17	3	0.333
	18	1	0.061
Risk factors, n			
Smoking	18	4	0.535
Hyperhomocysteinemia	4	1	0.385

a There were no significant differences between groups

**Table 2:** Comparison of serum lipid indicator levels among different groups [M(Q1,Q3)]

Variables	AS(*n* = 67)	Non-AS(*n* = 20)	Z	*P*
T-CHO, mmol/L	3.91(3.26,5.07)	3.15(2.80,4.49)^*^	-2.416	0.016
TG, mmol/L	1.18(0.86,1.62)	0.86(0.70,1.69)^**^	-2.820	0.005
HDL-C, mmol/L	1.06(0.88,1.28)	1.25(0.96,1.23)	-0.283	0.778
LDL-C, mmol/L	2.52(2.00,3.41)	2.10(1.91,2.58)^*^	-2.023	0.043
VLDL, mmol/L	0.49(0.31,0.64)	0.35(0.20,0.46)^*^	-2.149	0.032
ApoA1, g/L	1.09(0.94,1.31)	1.07(0.97,1.12)	-0.878	0.380
ApoB, g/L	0.85(0.63,1.15)	0.77(0.61,0.97)	-1.155	0.248
LP(a), mg/L	145.60(66.40,313.50)	58.70(26.30,183.10) ^*^	-1.997	0.046

^*^P < 0.05, ^**^P < 0.01

### LY86 is highly expressed in human tissues and is involved in the development of atherosclerosis

**Fig. 1:**
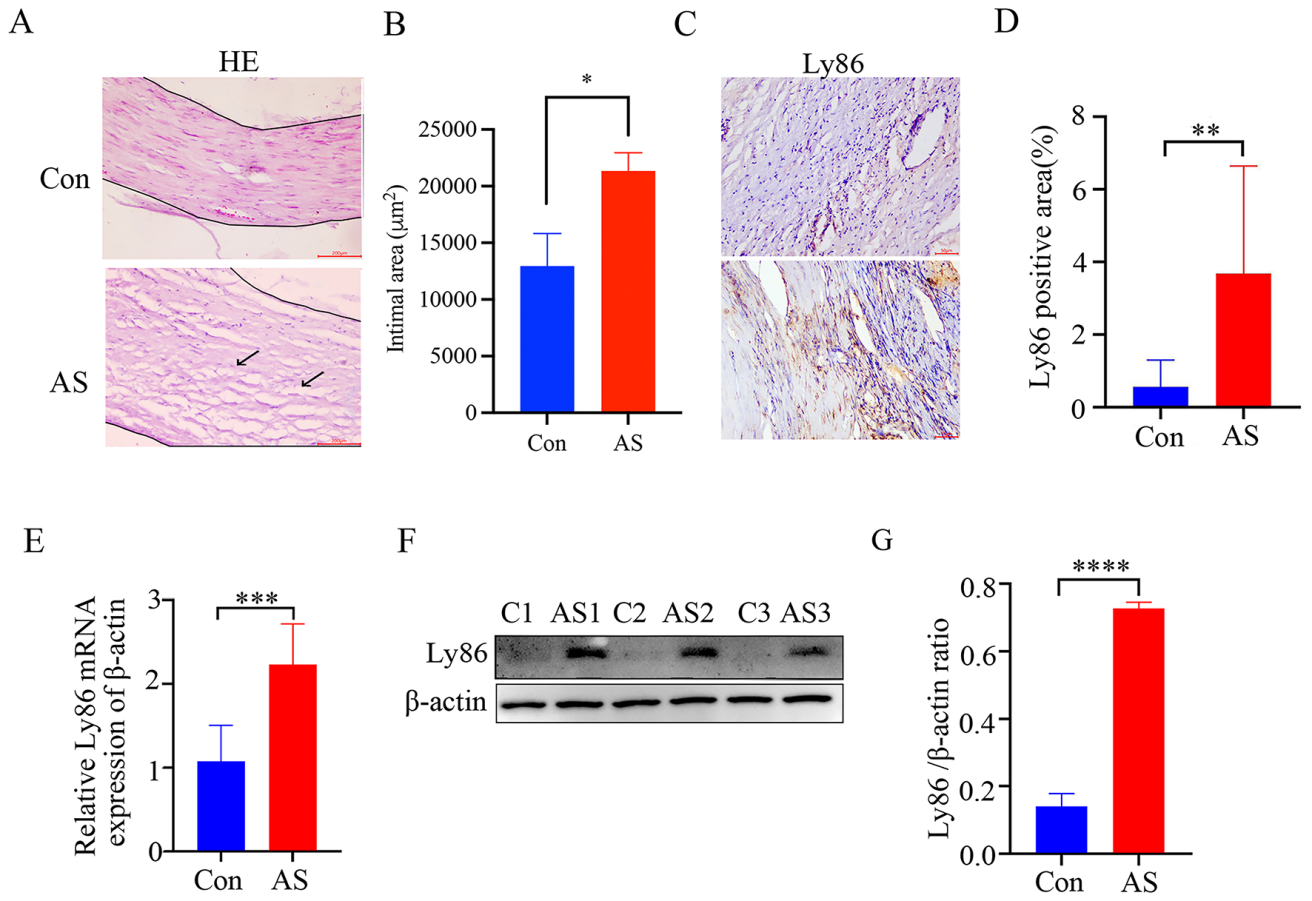
LY86 exhibits significantly elevated expression in human atherosclerotic tissues. (**A**,**B**) HE staining and statistical analysis of human carotid plaque tissue (×10) (*n* = 4). (**C**, **D**) Immunohistochemical staining and statistical analysis demonstrate the increased presence of LY86 in human carotid plaque tissue (×10) (*n* = 4). (**E**) Quantification of LY86 mRNA levels in human carotid plaque and adjacent normal intimal tissues (*n* = 6). (**F**, **G**) The cropped blot and quantification of LY86 protein in human carotid plaque and adjacent normal intimal (*n* = 3). The data are presented as mean ± SD; ^*^P<0.05, ^**^P<0.01, ^***^P<0.001. the cropped

**Fig. 2:**
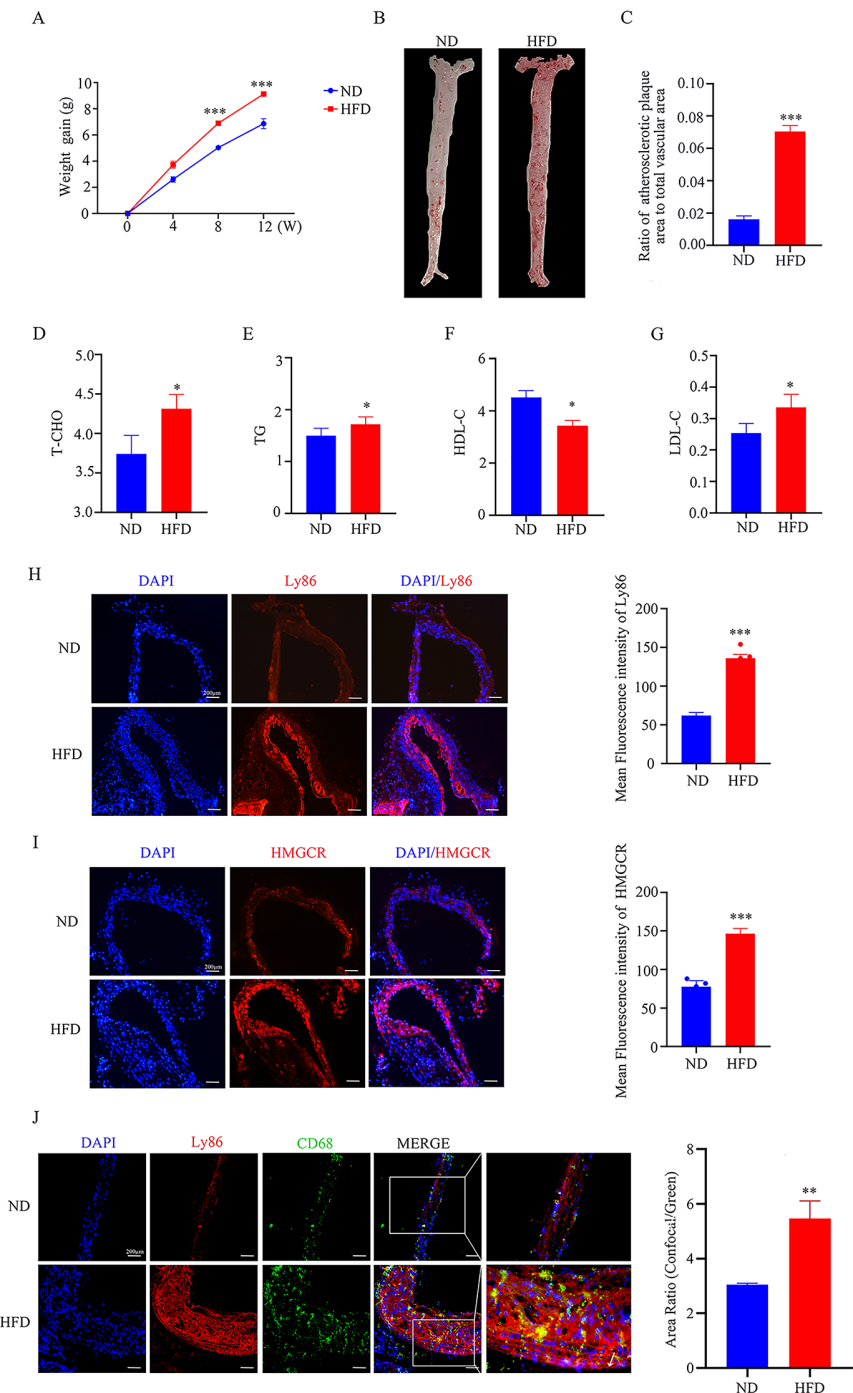
Ly86 expression is up-regulated in high-fat-induced atherosclerotic plaques in ApoE-/- mice. (**A**) Body weight statistics of mice at 0–12 weeks. (**B**,**C**) Mice thoracic and abdominal aortic tissue stained with oil red O. (**D**-**G**) Mouse Lipid Panel (T-CHO, TG, HDL-C, LDL-C). (**H**,**I**) Immunofluorescence staining of partial aortic arch in mice (LY86 and HMCGR). (**J**) The immunofluorescence co-localization of LY86 and CD68 in partial aortic arch of mice. The findings were presented as mean ± SD (*n* = 3); ^*^P<0.05, ^***^P<0.001

In order to investigate the expression of LY86 in atherosclerotic diseases, tissue samples were collected from patients undergoing carotid endarterectomy. The tissues underwent embedded sectioning and were subsequently subjected to HE staining and immunohistochemical staining. The results of the former revealed that intima in the control group appeared intact, well-organized, with densely packed nuclei and no evident damage; whereas, the intima exhibited significant thickening and increased plaque formation in the AS group (Fig. 1A, B). Immunohistochemical analysis demonstrated high expression levels of LY86 in the AS group (Fig. 1C, D). To further validate these findings, total protein and RNA were extracted from each tissue sample. Both Western blotting and qRT-PCR analyses consistently showed significantly elevated expression levels of LY86 in the AS group compared to the control group (Fig. 1E-G). Immunofluorescence co-localization studies showed that the LY86 and CD68, which is a macrophage marker, were was significantly up-regulated in the atherosclerotic plaques of ApoE ^−/−^ mice induced by high lipids (Fig. 2J).

### Ly86 expression is up-regulated in high-fat-induced atherosclerotic plaques in ApoE-/- mice

In the experiment, 20 male ApoE-/- mice were applied as research subjects, were randomly divided into experimental group (*N* = 10) and control group (*N* = 10), the experimental group was given high cholesterol diet, and the control group was given normal diet, and the body weight of the mice was recorded at the same time. At the end of modelling, the results showed that the body weight of ApoE-/- mice was significantly elevated compared to the control group and was statistically significant after 8 weeks (Fig. 2A). Thoracic and abdominal aortic tissues were stained with oil red O to confirm the success of membrane creation (Fig. 2B, C), and the four indices of blood lipids were detected in the mice, and the ApoE-/- mice showed a significant increase in body weight compared with the control group (Fig. 2D-G). Immunofluorescence staining of pathological sections of selected aortic arches showed that the expression of LY86 and HMCGR was upregulated in atherosclerotic plaques of ApoE-/- mice induced by high lipids (Fig. 2H, I).

### Construction of THP-1 macrophage lipid-loaded model and screening of LY86 knockdown sequence

**Fig. 3:**
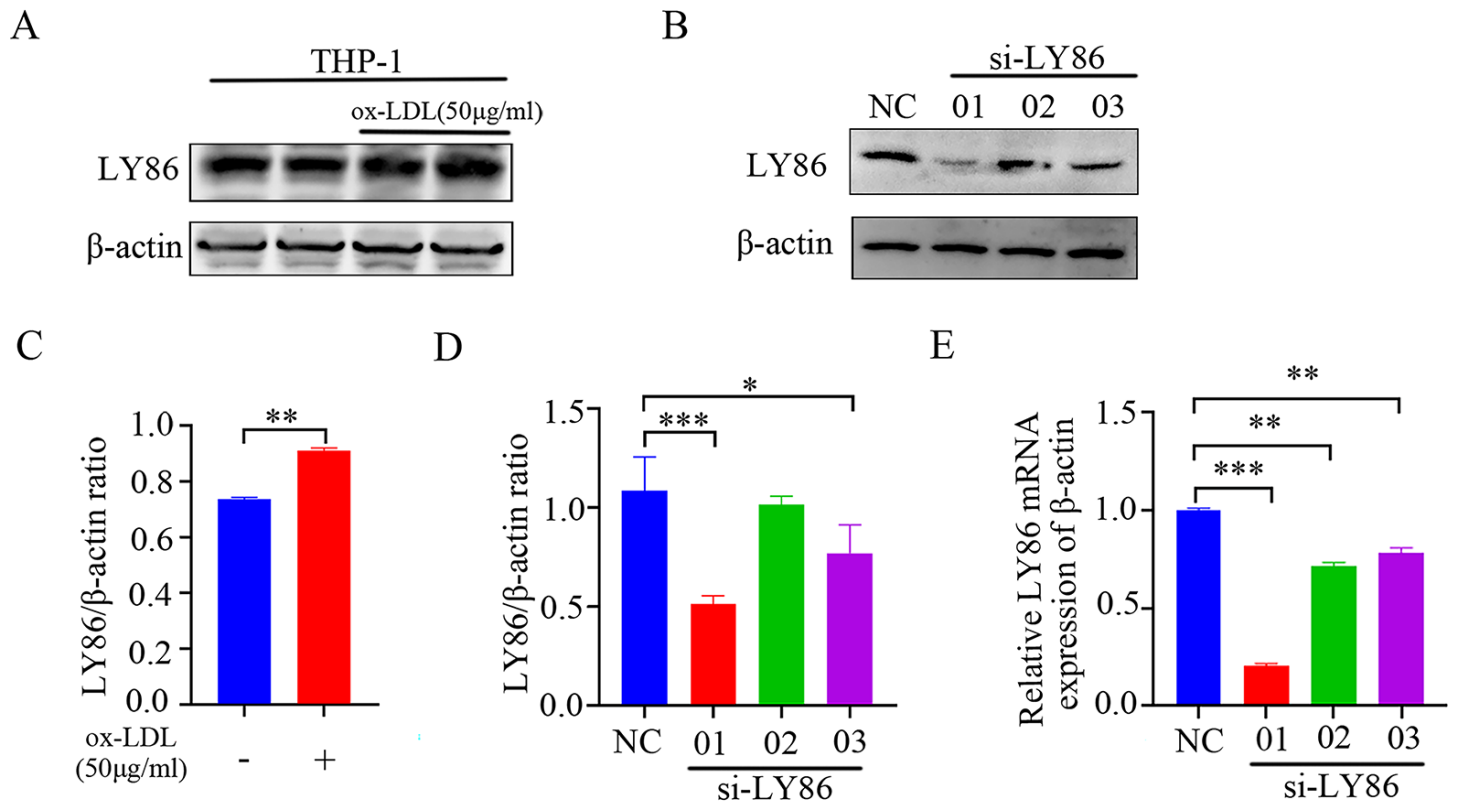
Construction of a THP-1 lipid-loaded model and screening of the LY86 knockdown sequence were performed. THP-1 cells were treated with 50 µg/ml ox-LDL for 24 h, and the cropped blot and quantification of LY86 protein. (Figures **A** and **C**). The expression level of LY86 was also evaluated by Western blotting and qRT-PCR after transfection of THP-1 cells with si-LY86-01 sequence for 24 h (Figures **B**, **D**, **E**). The results are presented as mean ± SD (*n* = 3); ^*^P<0.05, ^**^P<0.01, ^***^P<0.001

After treating THP-1 cells with ox-LDL (50 µg/ml) for 24 h, Western blot analysis revealed a significant increase in LY86 protein expression (Fig. 3A, C). Subsequently, the most effective siRNA targeting LY86 was selected for further experiments based on joint confirmation using Western Blot (Fig. 3B, D) and qRT-PCR (Fig. 3E and supplemental Fig. 1E, F).

### Silencing LY86 reduces ox-LDL-induced lipid accumulation in THP-1 macrophages

**Fig. 4:**
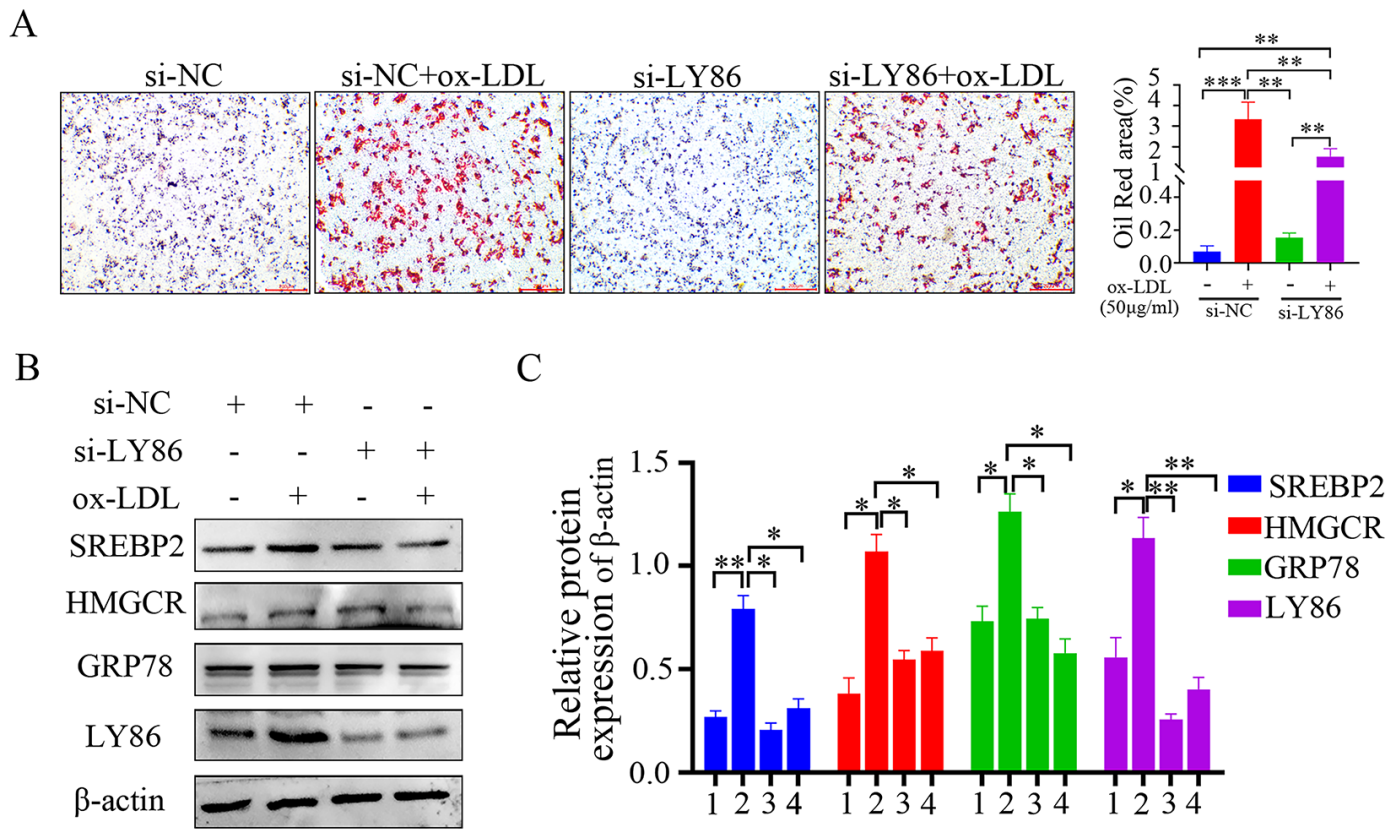
LY86 effectively attenuates ox-LDL-induced lipid accumulation in THP-1 macrophages. (**A**) Representative images of oil red O staining in THP-1 cells (×40 magnification). (**B**, **C**) The cropped blot and quantification of intracellular HMGCR, SREBP2, GRP78, and LY86 after 24-hour knockdown treatment with si-LY86 followed by 24-hour stimulation with ox-LDL; groups 1–4 represent the following conditions: 1 si-NC; 2 si-NC + ox-LDL; 3 si-LY86; and 4 si-LY86 + ox-LDL. Data are presented as mean ± SD (*n* = 3); ^*^P<0.05, ^**^P<0.01, ^***^P<0.001

The results of oil red O staining revealed that in the lipid-loaded state, a greater number of red lipid droplets were observed in the cells of the ox-LDL group compared to the control group. Upon silencing LY86 expression and subsequent ox-LDL treatment, intracellular lipid droplets were significantly reduced as compared to the ox-LDL group. This suggests that silencing LY86 can alleviate ox-LDL-induced lipid accumulation in THP-1 macrophages (Fig. 4A). Additionally, protein expression levels of SREBP2 and HMGCR in THP-1 macrophages were investigated after LY86 silencing and 24-hour ox-LDL treatment. Western blot analysis showed significant increases in protein levels of SREBP2, HMGCR, and GRP78 within the ox-LDL group when compared to controls; however, upon LY86 silencing followed by ox-LDL treatment, both protein levels decreased significantly thereby inhibiting processes associated with lipid accumulation (Fig. 4B, C). Futhermore, we also detected the expressions of M1 macrophage polarization marker CD86, matrix metalloproteinase9 (MMP9) and ATP binding cassette transporter A1 (ABCA1). MMP9, like M1 macrophages, is one of the important pro-inflammatory factors. ABCA1 is an important regulator of cholesterol efflux, which can inhibit the inflammatory reaction and affect the occurrence and development of AS. Western blot analysis showed that compared with the controls, the protein levels of CD86 and MMP9 were significantly increased, while the protein levels of ABCA1 were significantly reduced within the ox-LDL group. However, upon LY86 silencing followed by ox-LDL treatment, this trend was reversed by ox LDL treatment (supplemental Fig. 1A-D).

3.6 The effect of endoplasmic reticulum stress inhibitor 4-PBA on the expression of SREBP2 and HMGCR protein in THP-1 macrophages induced by ox-LDL.

**Fig. 5:**
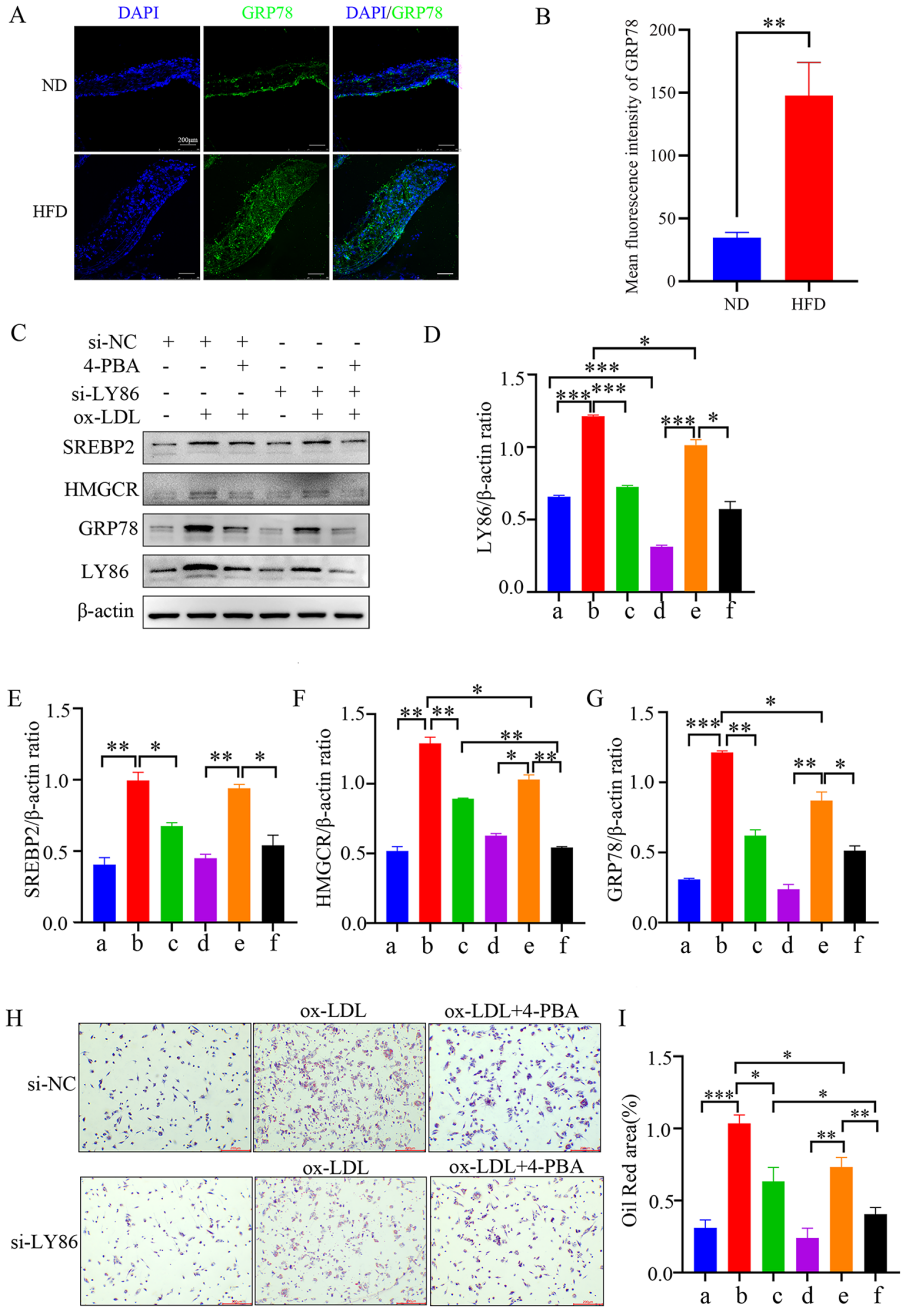
The effect of endoplasmic reticulum stress inhibitor 4-PBA on the expression of SREBP2 and HMGCR protein in THP-1 macrophages induced by ox-LDL. (**A**-**B**) Immunofluorescence staining of partial aortic arch in mice (GRP78). (**C**-**G**) The cropped blot and quantification of SREBP2, HMGCR, GRP78, and LY86 protein in THP-1 macrophages under the effect of 4-PBA on LY86 knockdown and ox-LDL. (**H**) THP-1 cells stained with oil red O (× 40). (**I**) Statistical analysis of Oil Red O staining results; a-f represent groupings, a si-NC, b si-NC + ox-LDL, c si-NC + ox-LDL + 4-PBA; d si-LY86, e si-LY86 + ox-LDL, f si-LY86 + ox-LDL + 4-PBA. The findings were presented as mean ± SD (*n* = 3); ^*^P<0.05, ^**^P<0.01, ^***^P<0.001

Several studies have also reported a correlation between endoplasmic reticulum stress and elevated cholesterol levels, and the inhibition of endoplasmic reticulum stress may impact lipid metabolism [17]. Immunofluorescence showed that the GRP78 expression was significantly up-regulated in the atherosclerotic plaques of ApoE -/- mice induced by high lipids (Fig. 5A-B). To investigate the role of LY86 in cholesterol metabolism, we employed the endoplasmic reticulum stress inhibitor 4-PBA. Western blot analysis revealed a significant suppression in GRP78 protein expression following 4-PBA treatment. In cells treated with ox-LDL (group c, group f), SREBP2 and HMGCR protein expression was notably lower compared to the untreated group (group a, group d); moreover, SREBP2 and HMGCR protein expression was significantly reduced in the si-LY86 knockdown group compared to the si-NC group as well (Fig. 5C-G). Additionally, cells were stained with oil red O after being subjected to different treatments (Fig. 5H, I). The results demonstrated that lipid accumulation corresponded consistently with the Western blot findings. These observations suggest that attenuation of endoplasmic reticulum stress may influence SREBP2 and HMGCR protein expression in THP-1 macrophages induced by ox-LDL.

## Discussion

In 2020, the global prevalence of increased intima-media thickness of the carotid artery is estimated at 27.6% and carotid stenosis is estimated at 1.5% among people aged 30 to 79 [18]. AS is a systemic disease that involves large arteries leading to stenosis and formation of atherosclerotic plaques. The cardiovascular disease caused by AS is the main cause of death and disability in human beings [19]. AS is the main pathological process of most CVDs, which can start in the early stage of life, latent and asymptomatic for a long time, and then develop to the late stage [20]. Carotid AS is the early manifestation of systemic AS. Therefore, in-depth exploration of the molecular mechanism of carotid AS is very important for the study of targeted drugs.

Zhao et al. found that the LY86 gene was highly expressed in carotid AS, and the higher the LY86 gene is, the worse the prognosis [21]. This study also found that the LY86 gene was highly expressed in carotid AS.

As an integral component of the plasma membrane lipids, cholesterol is a hydrophobic sterol molecule synthesized by all nucleated cells in the human body [22]. However, excessive levels of cholesterol can induce vascular damage. Therefore, maintaining optimal cholesterol levels is crucial for cardiovascular health. Cellular cholesterol metabolism encompasses uptake, biosynthesis, and efflux processes [23]. Cholesterol is transported via low-density lipoprotein (LDL), which interacts with LDL receptor (LDLR) or Niemann-Pick C (NPC) proteins on the cell membrane surface to facilitate cellular internalization [24]. In addition, it can also be internalized by scavenger receptors such as CD36 in myeloid cells [25]. HMG-CoA reductase (HMGCR) plays a crucial role in regulating intracellular cholesterol synthesis [26]. The efflux and transport of cholesterol between different organelles are mediated by ATP-binding cassette transporter 1 (ABCA1) or ATP-binding cassette subfamily G member 1 (ABCG1), ensuring a balanced cholesterol content across organelles [27, 28]. To prevent excess cholesterol toxicity, it must either be stored as cholesterol esters through the action of acyl coenzyme A: cholesterol acyltransferase (ACAT) or exported out of the cell [29, 30]. Research has shown that macrophages play an important role in cholesterol metabolism. On the one hand, macrophages can uptake large amounts of ox-LDL from the endothelium of blood vessels through their surface scavenger receptors, then store them in cells in the form of cholesterol esters [31]. Macrophages can also export cholesterol ester to the extracellular space in the form of ApoA-1 and high density lipoprotein through ABCA1-mediated cholesterol efflux pathway [32]. However, with the continuous uptake of ox-LDL by macrophages, the rate of cholesterol inflow is greater than the rate of cholesterol efflux, which causes the breakdown of cholesterol metabolism balance. It resulted in excessive accumulation of cholesterol in the cells, and then trigger macrophage foaming and inflammation, ultimately leading to the formation of AS plaques and promoting the development of the disease [31]. Therefore, inhibiting macrophage foaming is of great significance to improve AS. In this article, We applied PMA to treat THP-1 monocytes and differentiated them into macrophages [15].Then, We applied ox-LDL to treat macrophages and constructed the lipid-loaded model. While, silencing LY86 could reduce the ox-LDL-induced lipid accumulation in macrophages.

However, the underlying mechanisms by which LY86 regulates macrophage cholesterol metabolism remain unclear. Some studies have also reported a link between endoplasmic reticulum stress and high cholesterol, and the inhibition of endoplasmic reticulum stress may affect lipid metabolism levels [17]. Recently, Endoplasmic reticulum (ER) stress has emerged as a pivotal underlying mechanism in the pathogenesis and progression of non-alcoholic fatty liver disease (NAFLD) and non-alcoholic steatohepatitis (NASH) [33]. It is now widely recognized that both hyperglycemia and lipid accumulation can disrupt proteostasis, leading to ER stress [34, 35]. Various pathological and physiological events perturb these mechanisms, resulting in protein misfolding/unfolding and heightened ER stress. The consumption of a high-fat diet (HFD) has also been implicated in inducing hepatic ER stress [36]. Depending on the stage of the disease, atherosclerosis induces increased production of reactive oxygen species (ROS) and triggers an ER stress response. An analysis involving 152 autopsy samples from human coronary arteries was conducted to ascertain the significance of GRP78 and CHOP expression induction, as well as elucidate the relationship between ER stress and atherosclerosis [37]. In this article, we observed that the expression of GRP78 protein in macrophages treated with ox-LDL and ApoE-/- mice, indicating ER stress in macrophages. Subsequently, we investigated the intricate association between LY86 and ER stress to elucidate a novel mechanism by which LY86 modulates lipid deposition in macrophages. We applied the ER stress inhibitor 4-PBA. 4-PBA is a low molecular weight, non-toxic fatty acid, whih has been found to have chaperone like activity. It can reduce the number of mutated or unfolded proteins retained in cells during ER stress and act as a molecular chaperone to exert anti-inflammatory effects [38]. This experiment observed a small amount of granular lipid deposition in the control group macrophages after stained with oil red O. However, a large amount of granular lipid deposition was observed in macrophages treated with ox-LDL, meanwhile, the expression of LY86, SREBP2, and HMGCR proteins was significantly increased. The ox LDL + 4-PBA 4 group of si-LY86 silenced macrophages showed a significant reduction in lipid particles, with only partial granular lipid deposition. At the same time, the expression of SREBP2 and HMGCR were also significantly reduced.

In addition to its involvement in LY86-induced lipid metabolism, ER stress has been reported to play a role in the ROS/MAPK signaling pathway. Deficiency of LY86 exacerbates autophagy through activation of the ROS/MAPK signaling pathway [39]. This raises the question of whether LY86 can also impact the ROS/MAPK signaling pathway in macrophages, which requires further investigation. Currently, statins are primarily used for treating hyperlipidemia; however, their therapeutic efficacy remains limited for severe patients [40, 41]. Therefore, it is imperative to identify novel drug targets. This article aims to discover new therapeutic targets and provide fundamental research for clinical treatment of cardiovascular diseases.

## Conclusion

This study demonstrates the involvement of LY86 in atherosclerosis development, with high expression observed in human plaque tissues. Mechanistic investigations reveal that LY86 mediates ox-LDL-induced macrophage lipid accumulation through the SREBP2/HMGCR pathway.

### Electronic supplementary material

Below is the link to the electronic supplementary material.

Supplementary Material 1

Supplementary Material 2

## Data Availability

The data of this study are available on request from the corresponding author (Wei Bi), uponreasonable request.
