# Supplementary figures and images for: LY86 facilitates ox-LDL-induced lipid accumulation in macrophages by upregulating SREBP2/HMGCR expression
Source: BMC Cardiovasc Disord. 2024 May 31;24:289. doi: 10.1186/s12872-024-03957-1 (PMC11140969; doi:10.1186/s12872-024-03957-1)

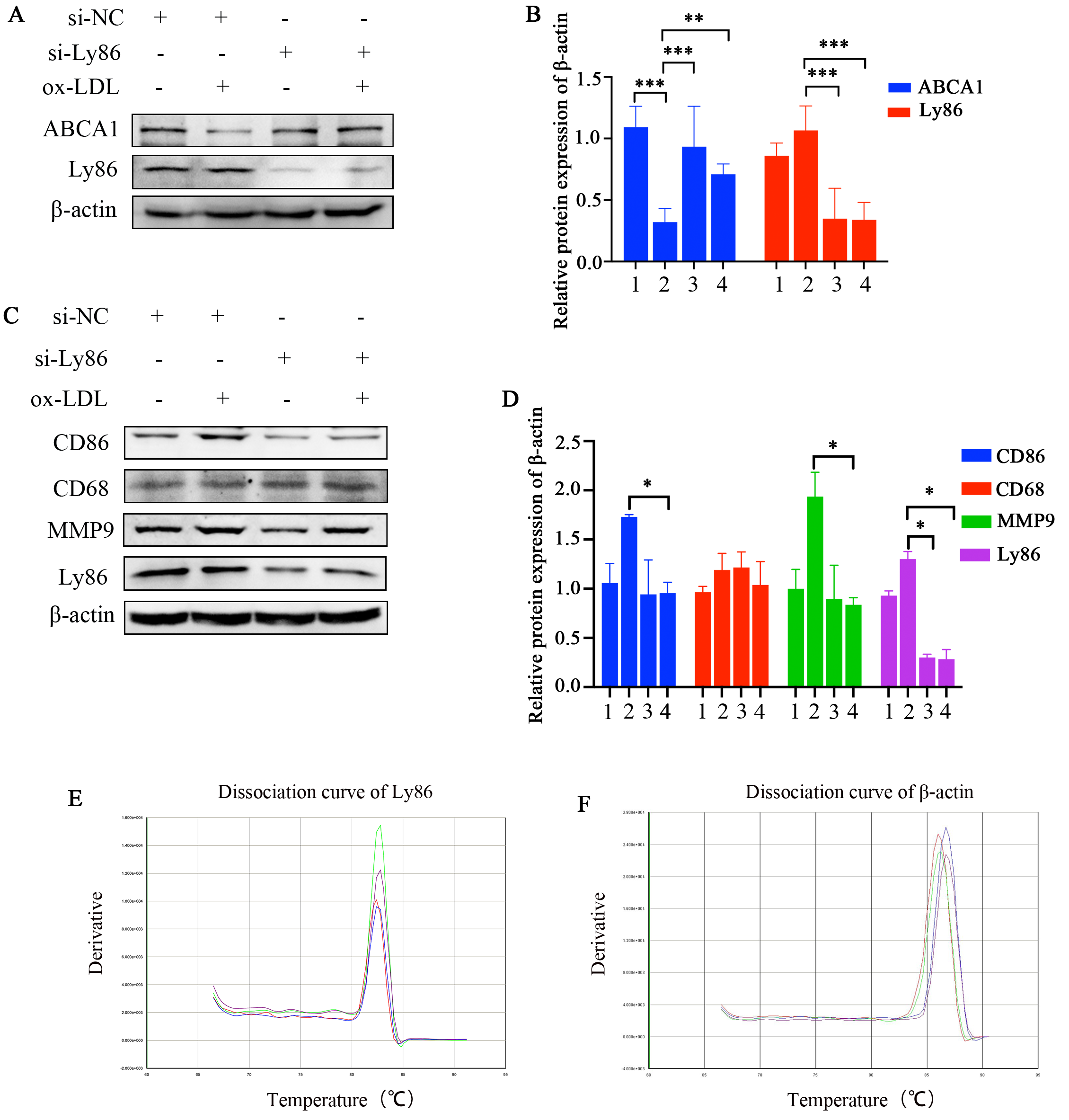

Supplement: Supplementary file 1 — Supplementary Material 1 [file 12872_2024_3957_MOESM1_ESM.tif]
