# Supplementary material for: LY86 facilitates ox-LDL-induced lipid accumulation in macrophages by upregulating SREBP2/HMGCR expression
Source: BMC Cardiovasc Disord. 2024 May 31;24:289. doi: 10.1186/s12872-024-03957-1 (PMC11140969; doi:10.1186/s12872-024-03957-1)

Fig.1E

β-actin
（42kD）

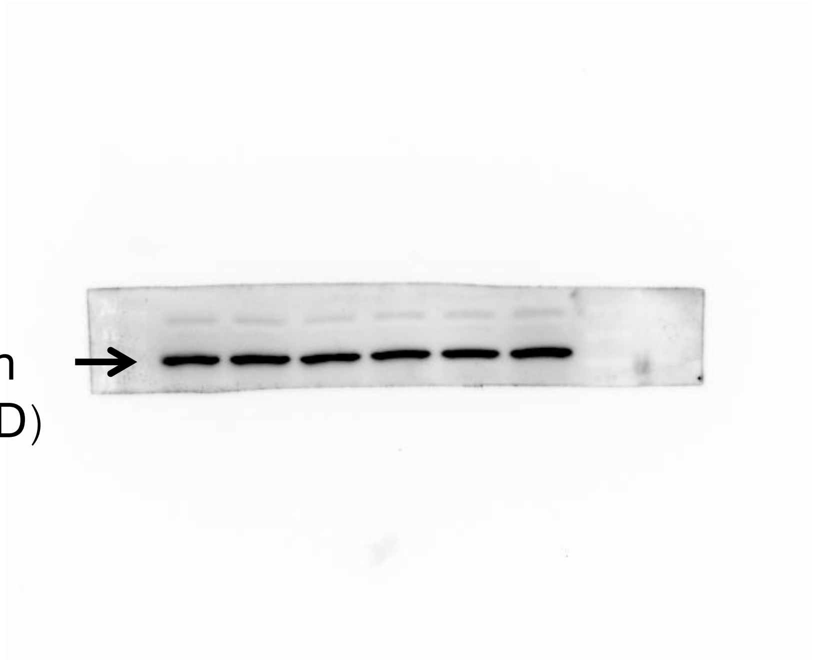

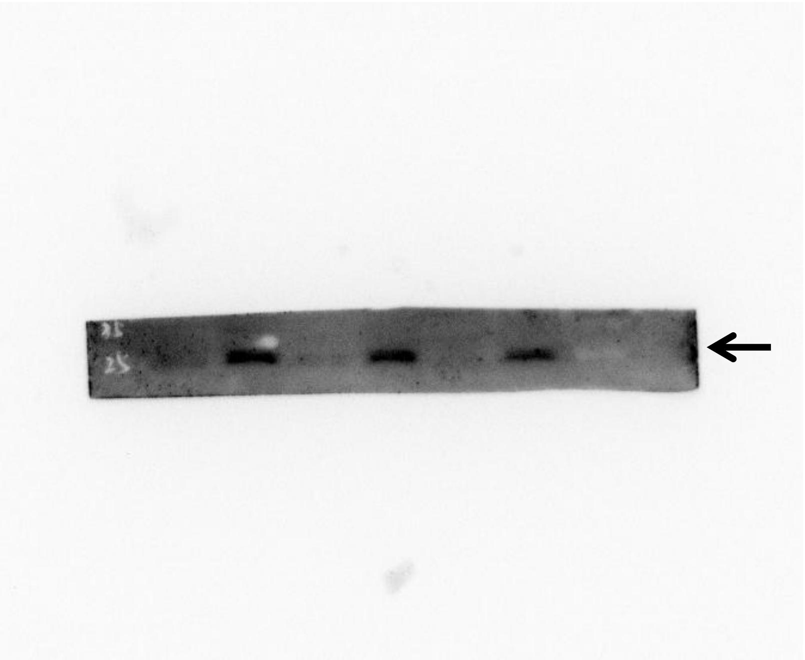

LY86
（26KD）

Fig.3A

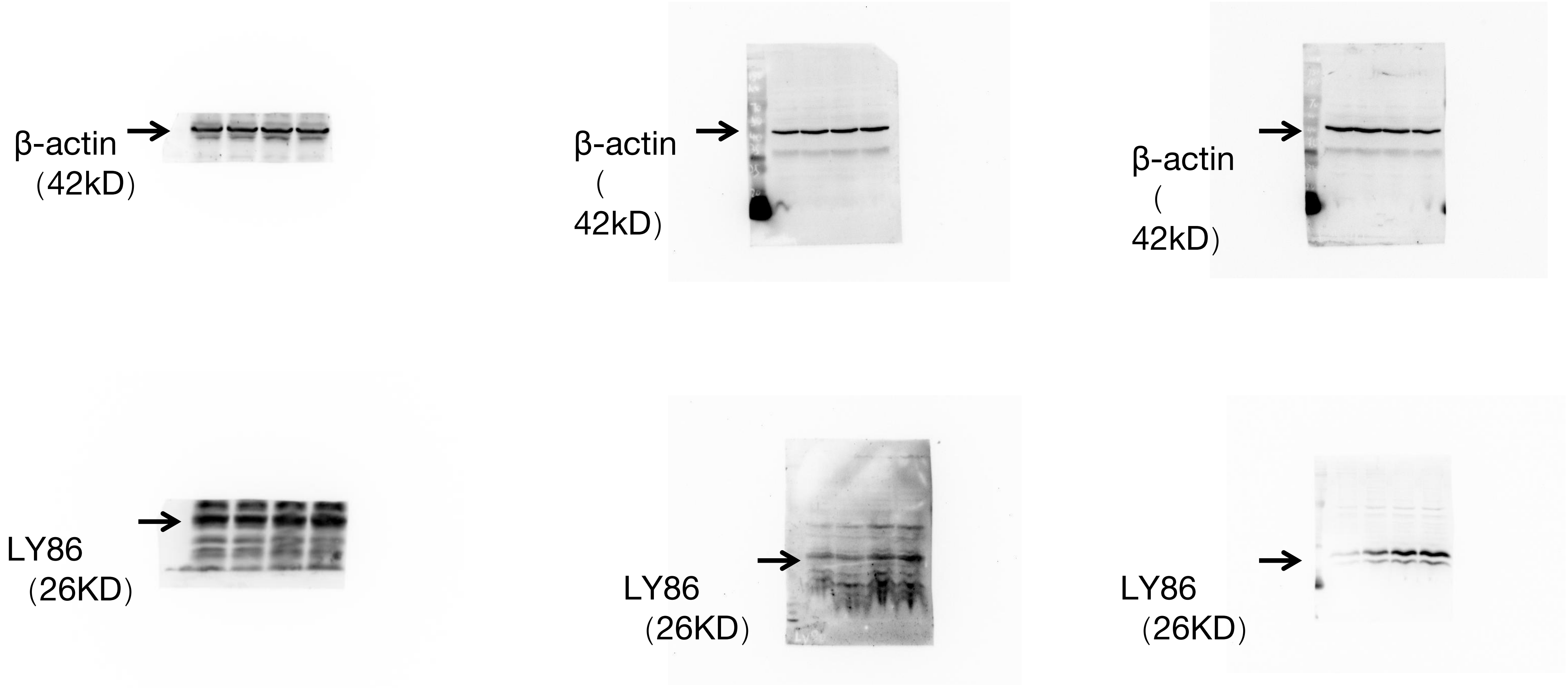
β-actin
(42kD)
β-actin
(
42kD)
β-actin
(
42kD)
LY86
(26KD)
LY86
(26KD)
LY86
(26KD)

Fig.3B

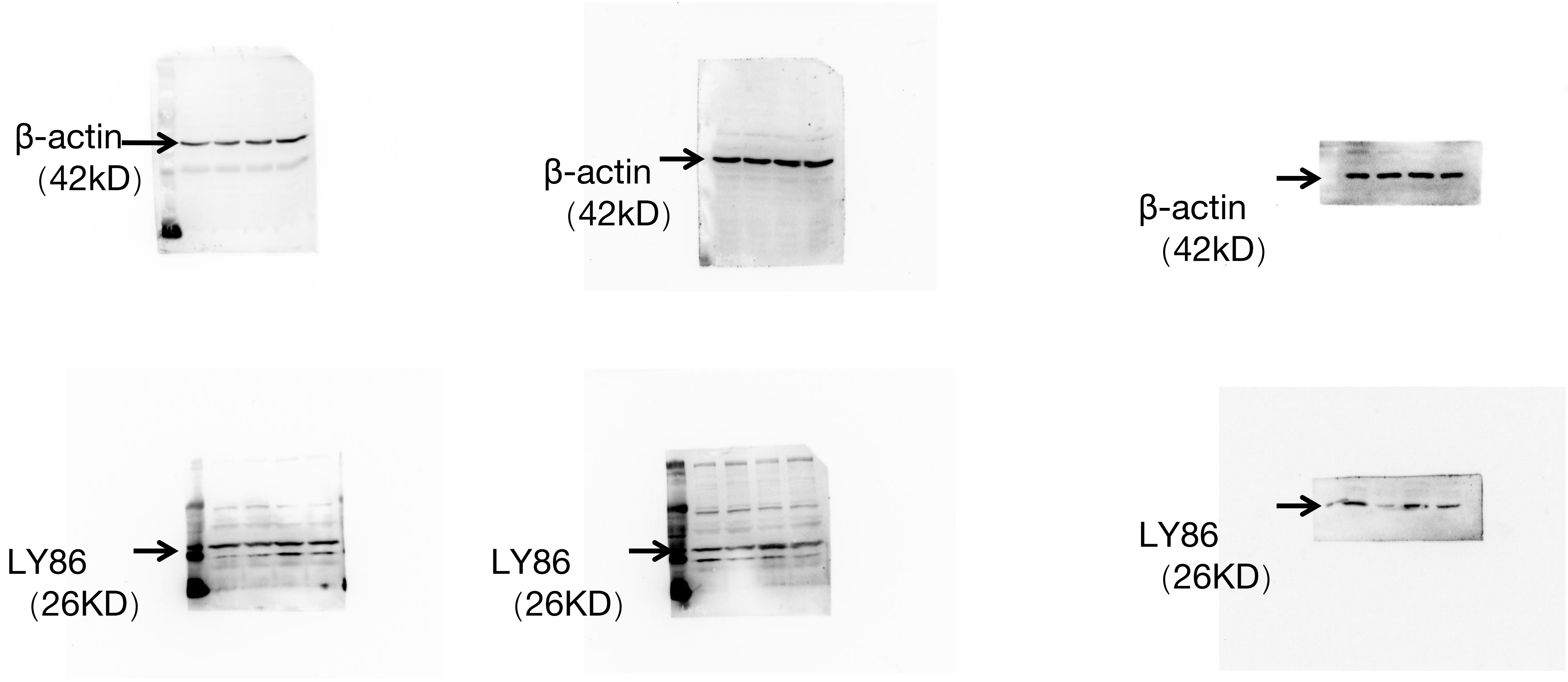
β-actin
(42kD)
β-actin
(42kD)
β-actin
(42kD)
LY86
(26KD)
LY86
(26KD)
LY86
(26KD)

Fig.4B(1)

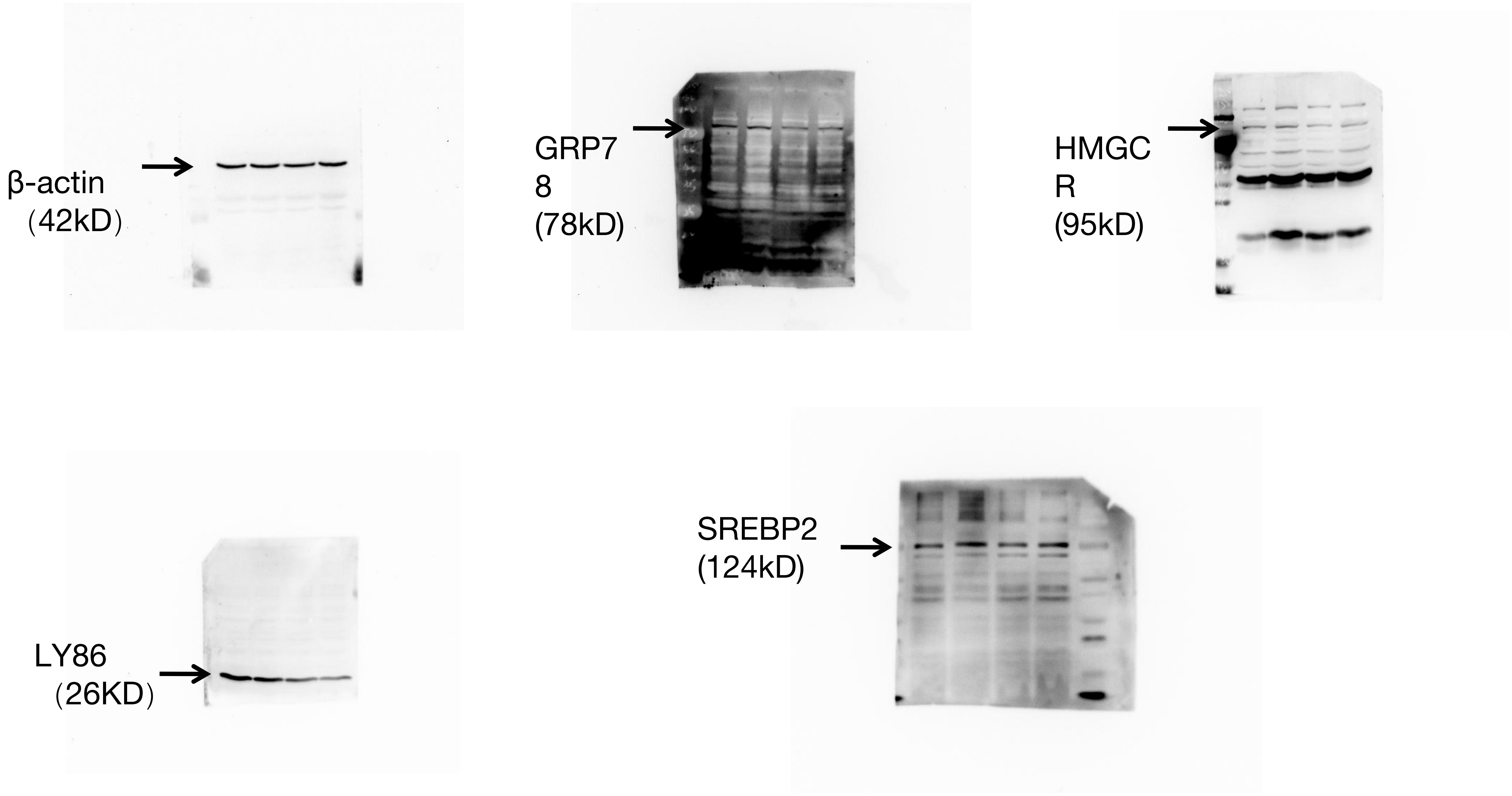

Fig.4B(2)

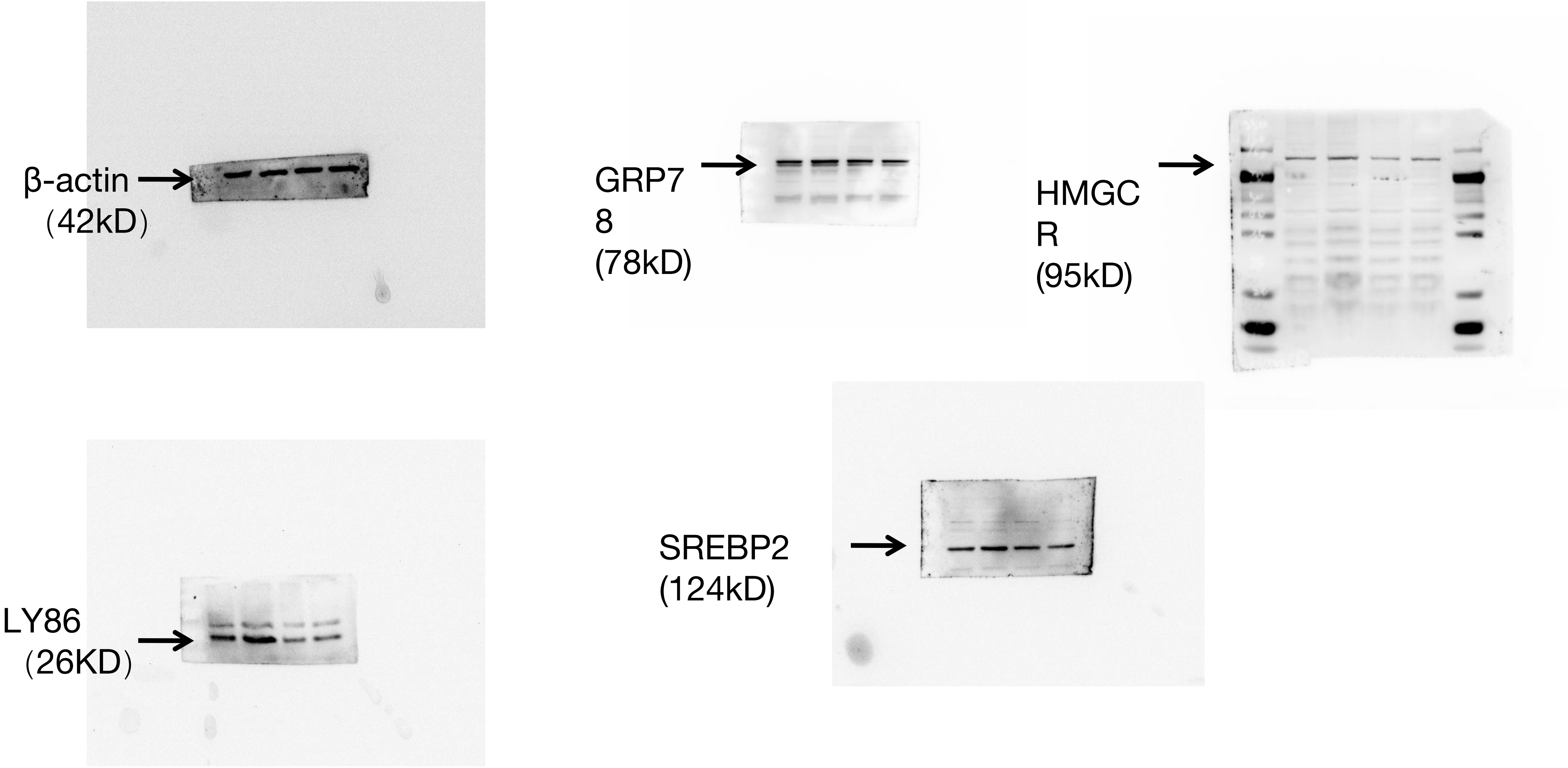

Fig.4B(3)

β-actin
(42kD)

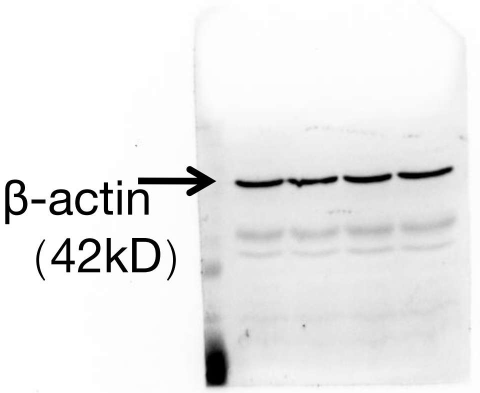

GRP7
8
(78kD)

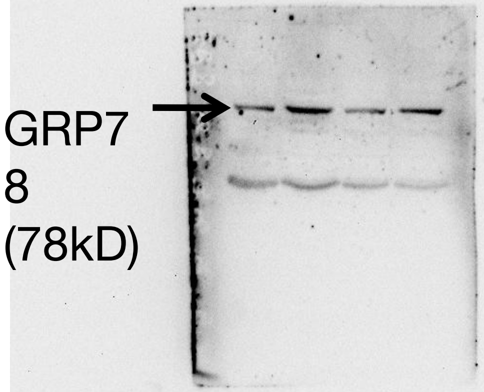

HMGCR
(95kD)

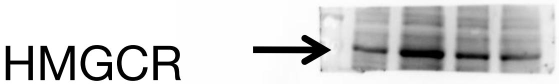

LY86
(26KD)

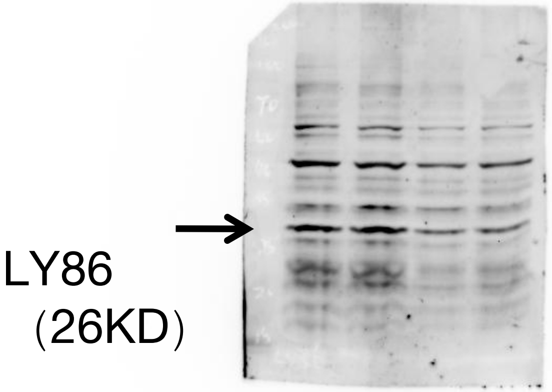

SREBP2
(124kD)

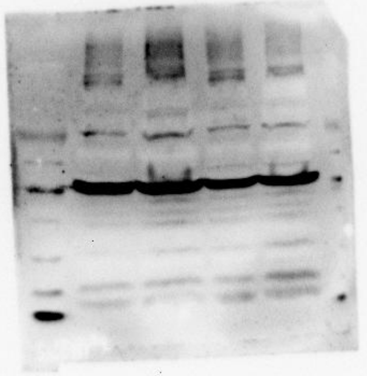

Fig.5C(对照文章)（1）

β-actin
（42kD）

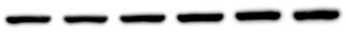

GRP7
8
(78kD)

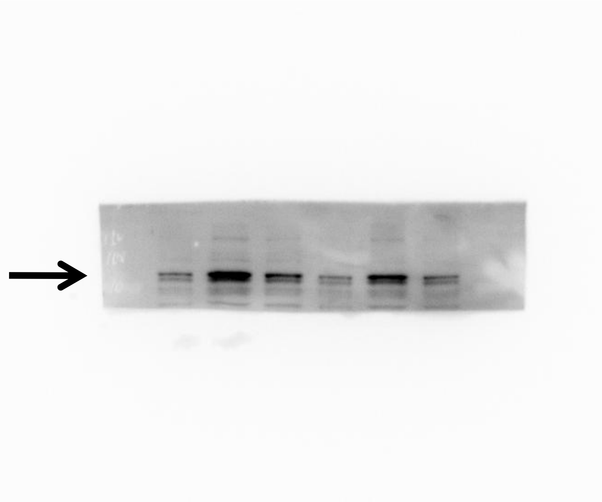

HMGC
R
(95kD)

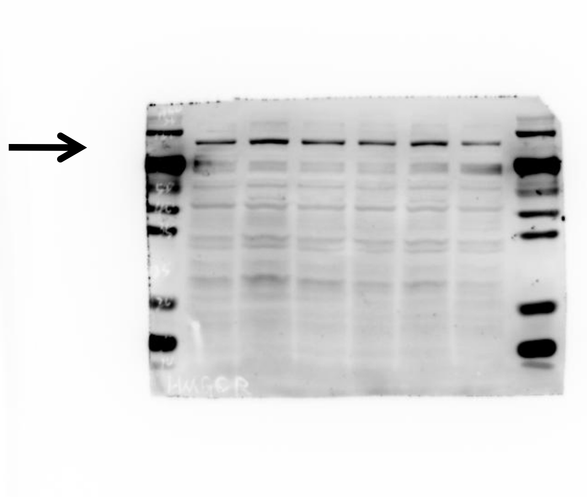

LY86
（26KD）

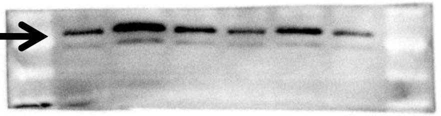

SREBP
2
(124kD)

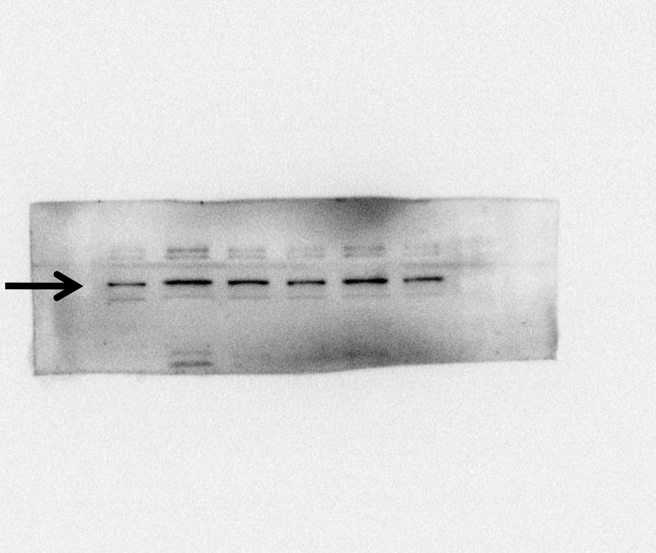

Fig.5C(2)

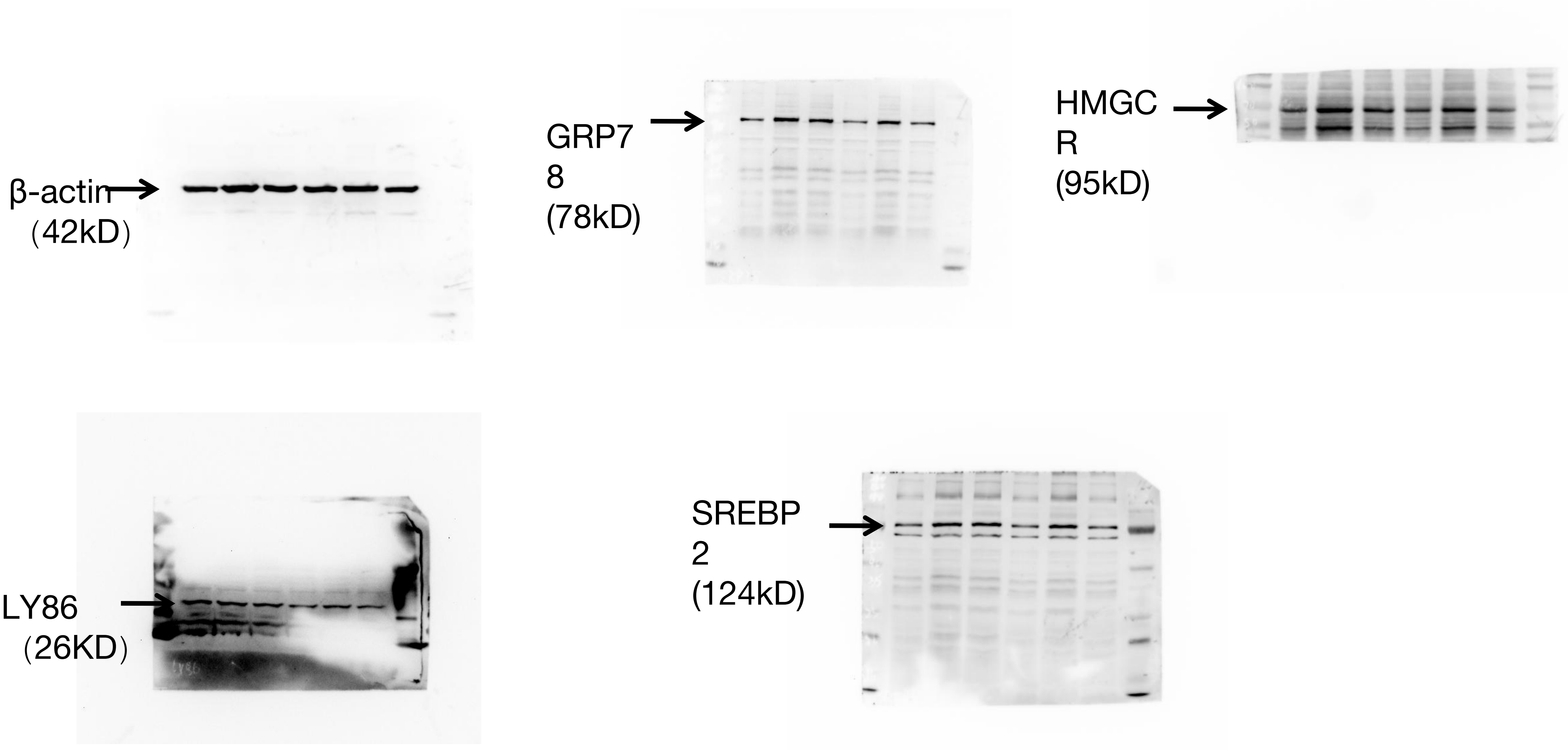

Fig.5C(3)

GRP7
8
(78kD)

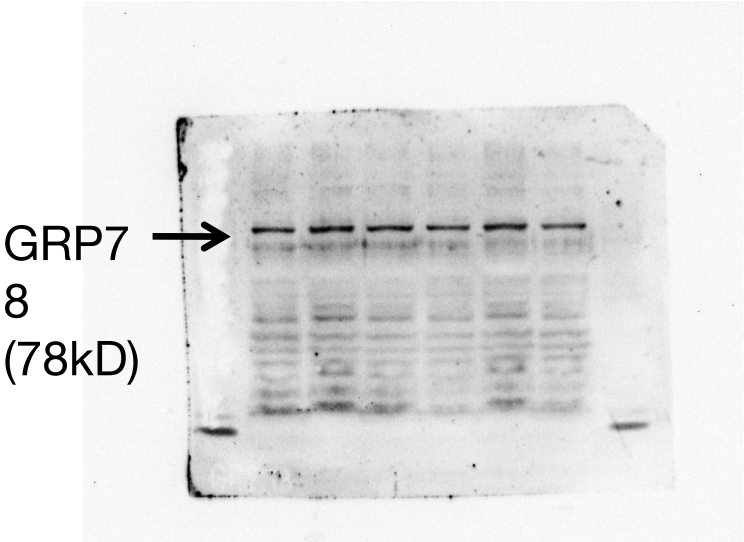

HMGCR
(95kD)
β-actin
(42kD)

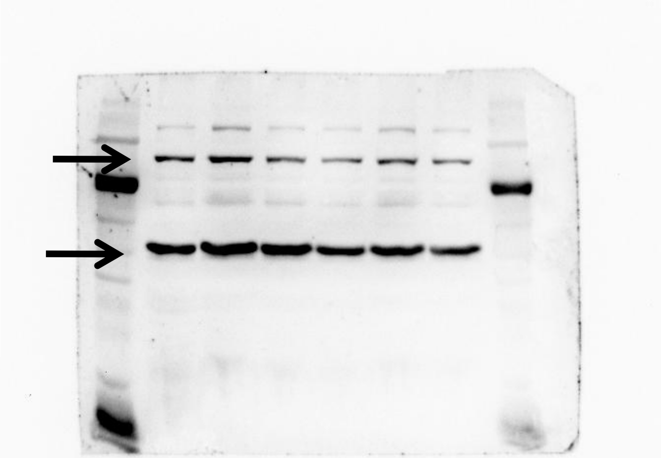

LY86
(26KD)

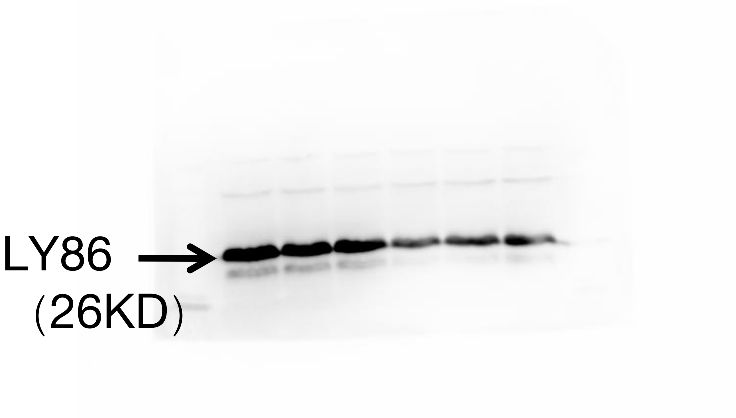

SREBP2
(124kD)

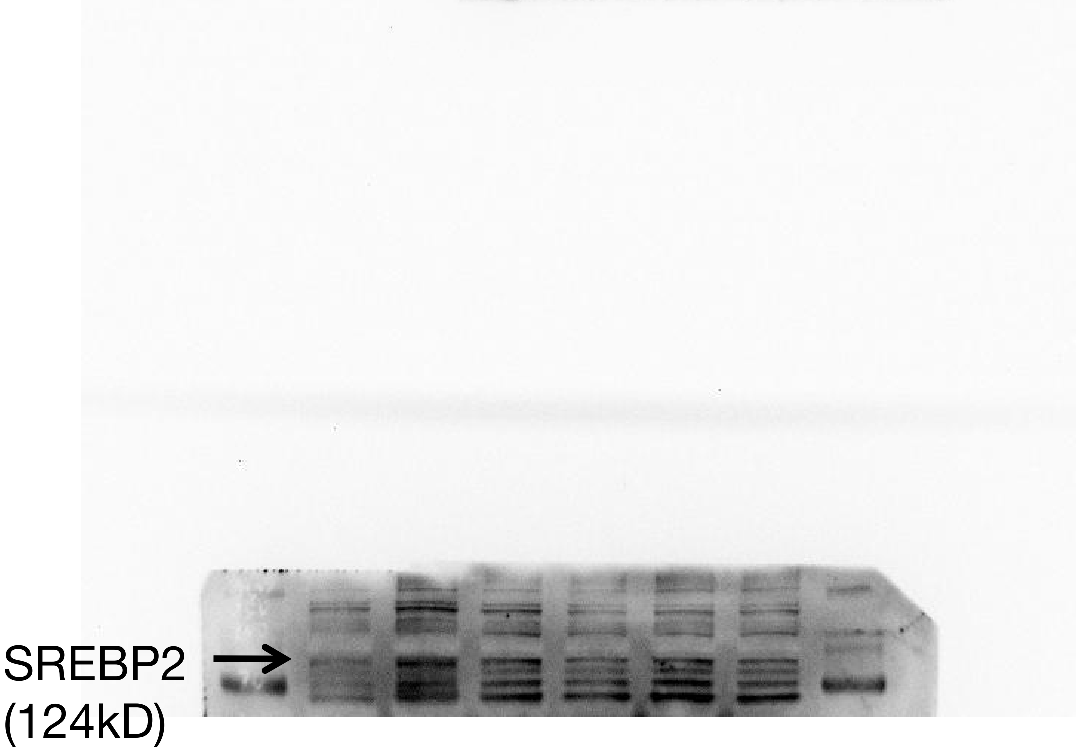

Fig.S1 A（1）

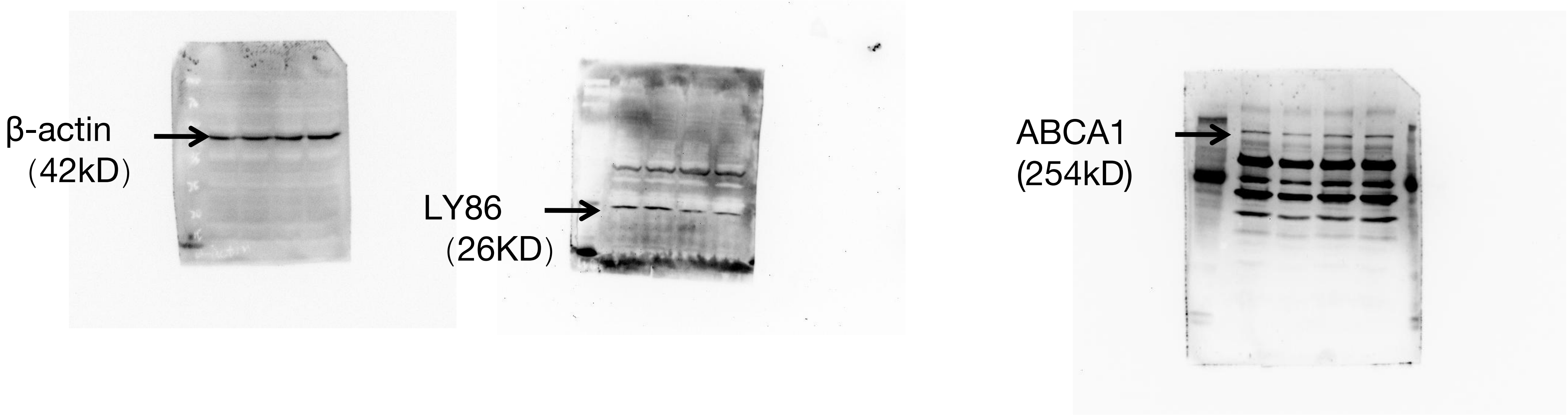

Fig.S1 A（2）

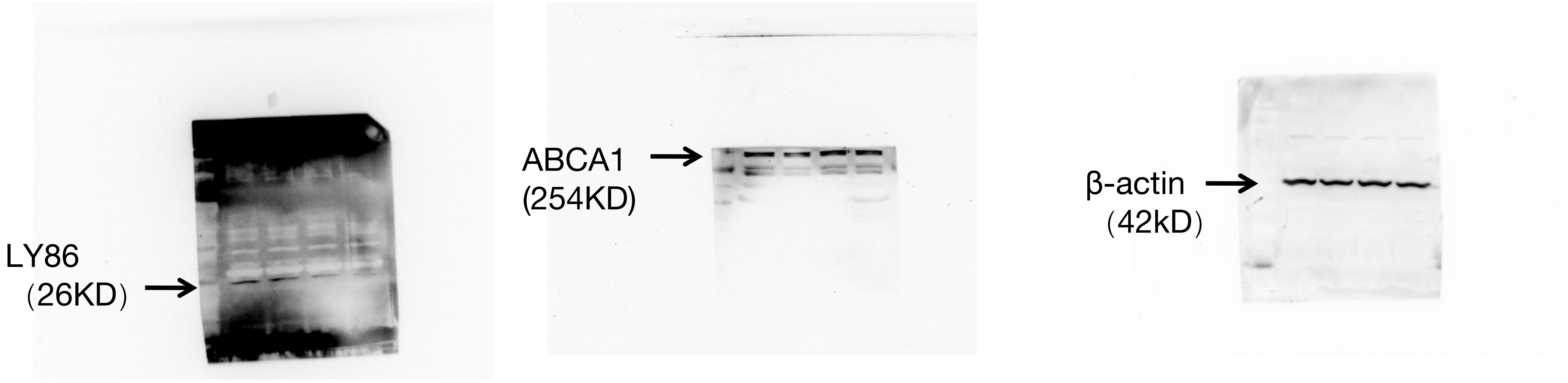

Fig.S1 A (3)

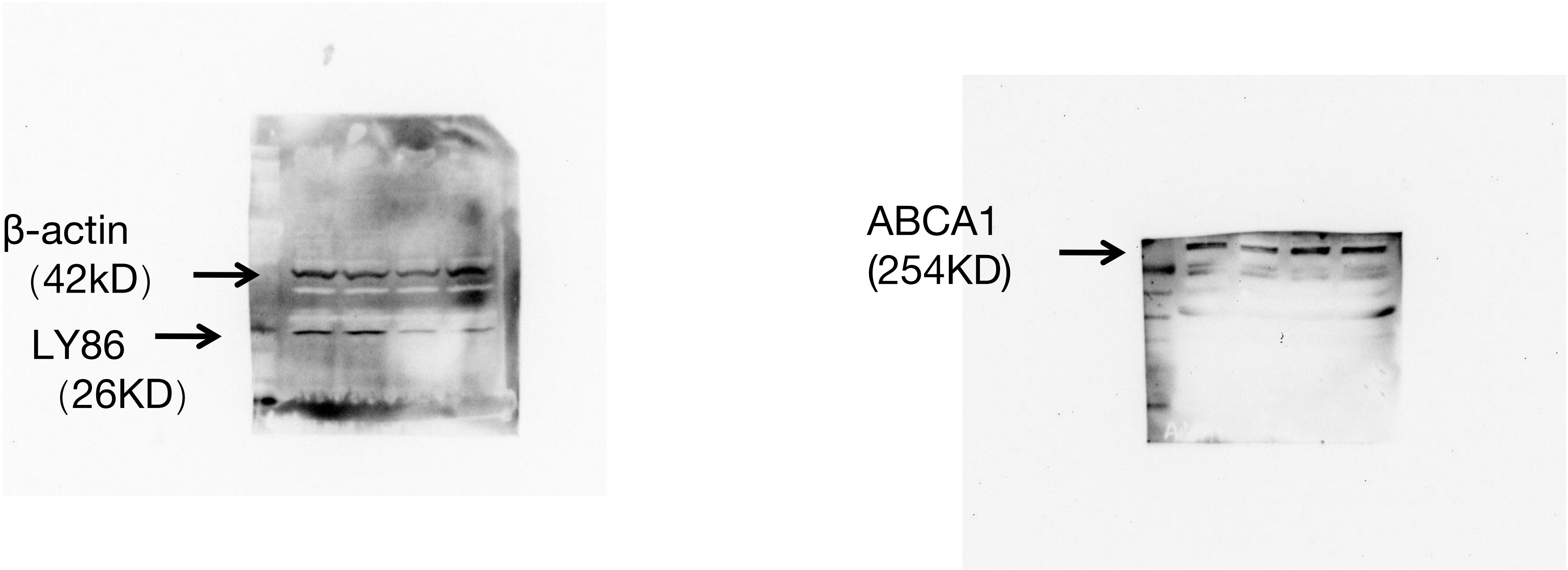

Fig.S1 C （1）

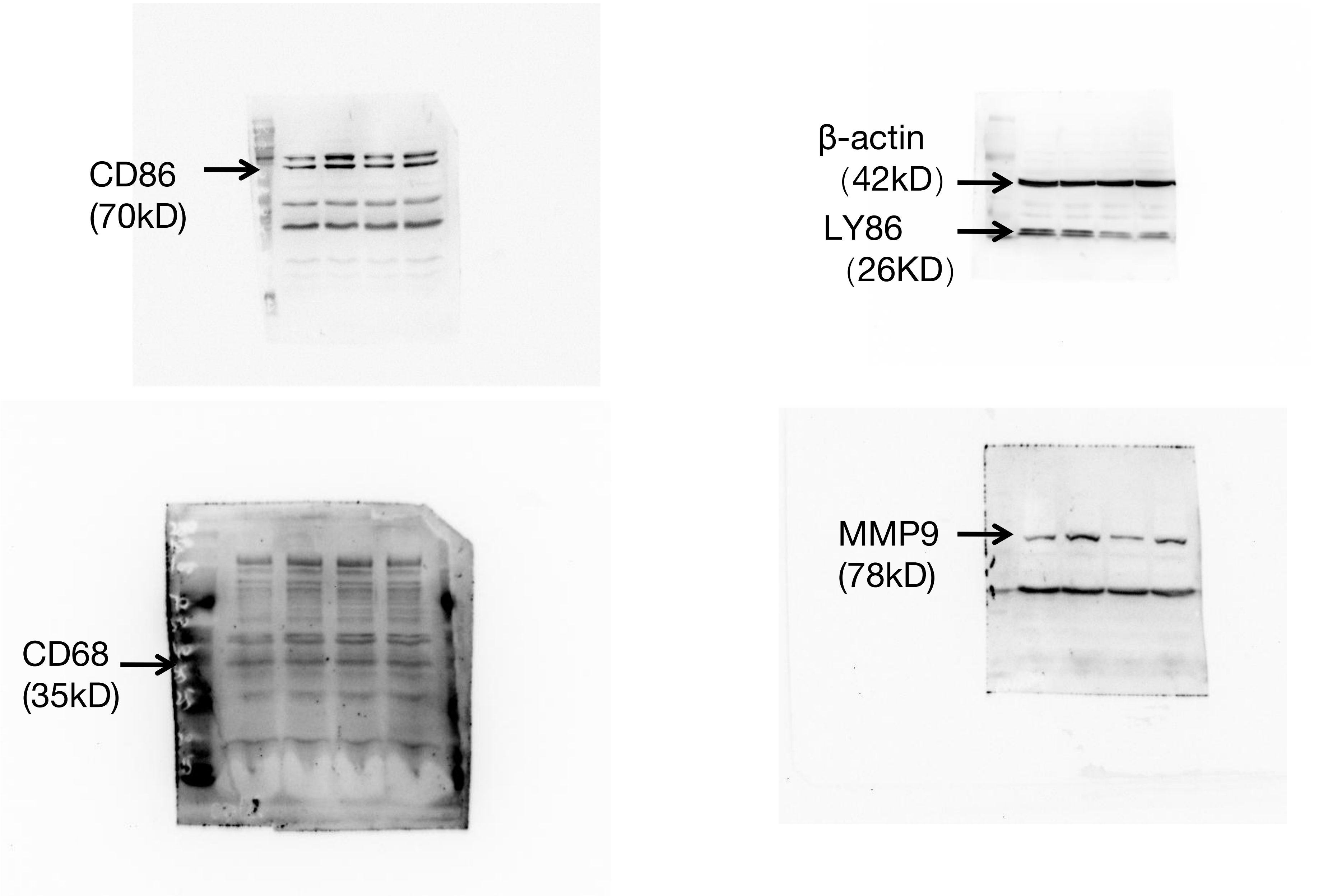

Fig.S1 C （2）

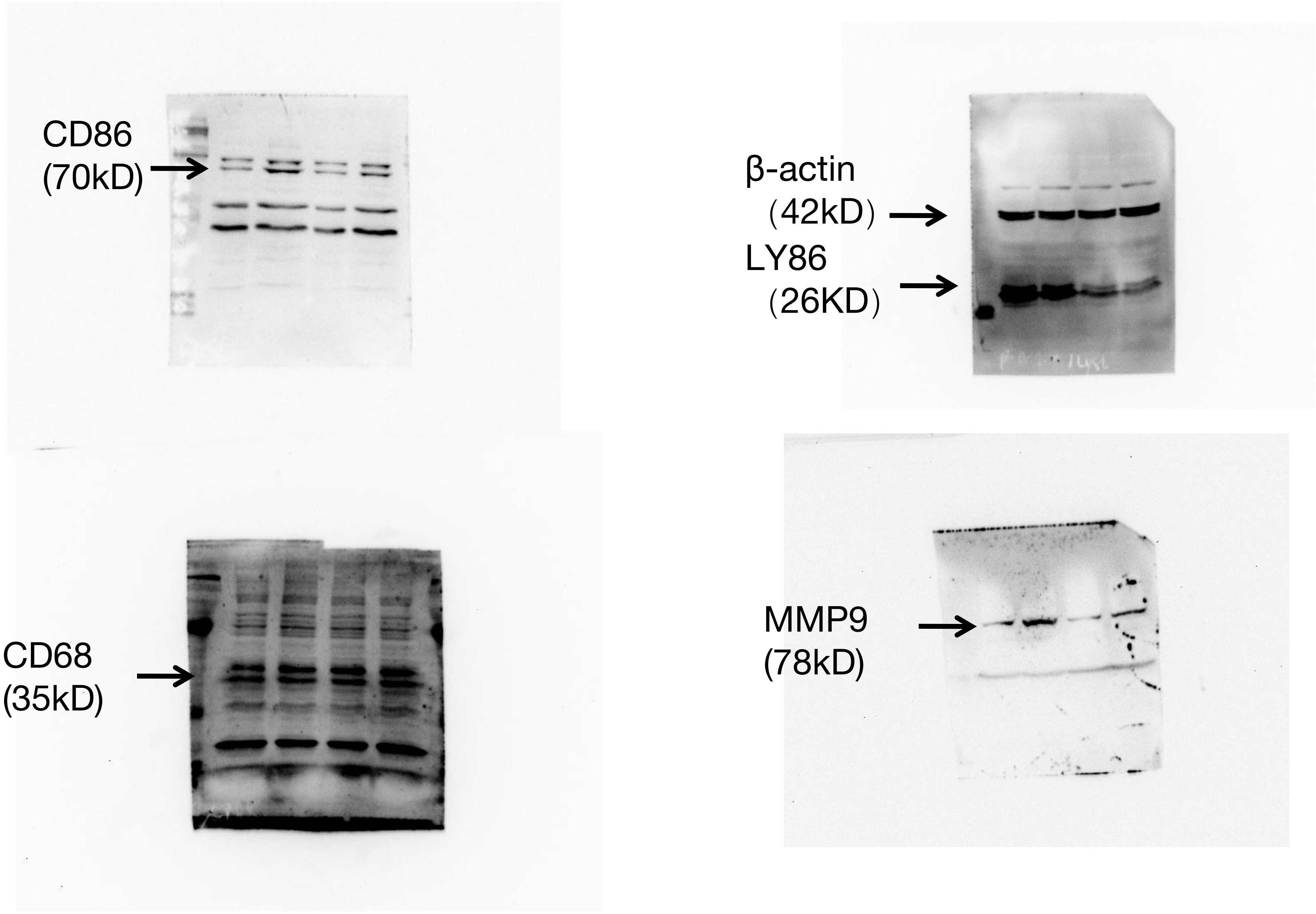

Fig.S1 C （3）

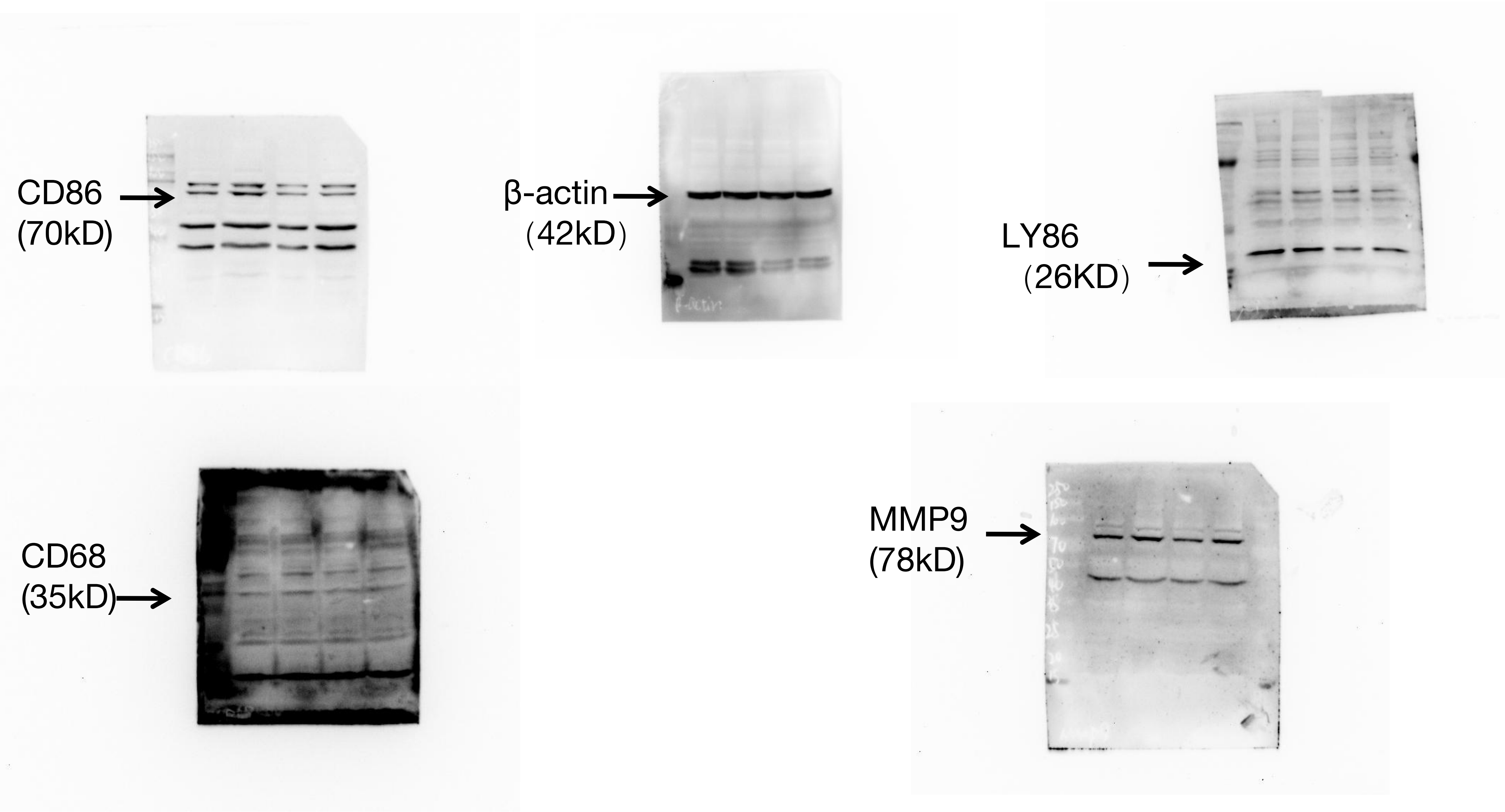

Supplement: Supplementary file 2 — Supplementary Material 2 [file 12872_2024_3957_MOESM2_ESM.pdf]
